# Timelines for returning to physical activity following pediatric spinal surgery: recommendations from the literature and preliminary data

**DOI:** 10.1186/s13104-021-05571-2

**Published:** 2021-04-29

**Authors:** Leanne R. Willson, Madeline Klootwyk, Laura G. Rogers, Kathleen Shearer, Sarah Southon, Christina Sasseville

**Affiliations:** 1 The King’s University, 9125–50 Street NW, Edmonton, AB T6B 2H3 Canada; 2 Stollery Children’s Hospital, 8440 112 St NW, Edmonton, AB T6G 2B7 Canada; 3 University of Alberta, Faculty of Nursing 4-141 Edmonton Clinic Health Academy, 11405 87 Ave, Edmonton, AB T6G 1C9 Canada

**Keywords:** Pediatrics, Spinal surgery, Physical activity, Following medical recommendations, Orthopedics, Surgeon recommendations

## Abstract

**Objective:**

Participation in physical activity and sports is known to have positive implications for physical health, and for social and emotional wellbeing of children. Following corrective spinal surgery for scoliosis, the timeline for the return to activities and sports varies from surgeon to surgeon and from location to location, and return to activities can be limited due to pain, fear, and decreased flexibility. It is critical that patients know best-practice guidelines, and it is equally critical that medical professionals know whether their patients are following those guidelines. This paper includes a summary of recommendations published in the literature, and a pilot study to address a gap in the literature on determining how long, post-surgery, adolescents with idiopathic scoliosis waited before returning to various self-care and physical activities, and what factors influenced return to activities. We used a mixed-method approach that involved two phases: a questionnaire (n = 8), and subsequent interviews of some participants (n = 3). Participants were ages 14–17 (M = 15.4) and had had posterior instrumentation and fusion for scoliosis in the past 2 years.

**Results:**

Some patients were cautious about return to activities, either because of emotional or medical reasons. However, in many instances, participants returned to physical activities earlier than was recommended, primarily for emotional and social reasons.

**Supplementary Information:**

The online version contains supplementary material available at 10.1186/s13104-021-05571-2.

## Introduction

Participation in sports and physical activity is essential for the psychosocial health and wellbeing of children and adolescents [1]. Children diagnosed with scoliosis may participate in sports and activities at the same rate as their peers; if surgical intervention is required, post-operative patients can show a decrease in physical activity due to the loss of flexibility and the experience of pain [2]. This may result in necessary restrictions or limitations on their participation in activities, which can have negative impacts on the psychosocial wellbeing of patients due to the inability to participate in regular activities like sports and gym classes [2].

Following surgery for adolescent idiopathic scoliosis (AIS), participation guidelines are important for guiding children and their parents toward or away from various activities. While there are immediate post-surgery consensus guidelines from surgeons prior to scoliosis patients leaving the hospital [3], there are no documented comprehensive exercise and participation guidelines for physicians to give patients post-operatively [4, 5]. The return to athletics and physical activity is largely dependent on the philosophy of the specific surgeon [6] based on the time since surgery, the instrumentation used, and the specific sport [7]. “As in all areas of medicine where there is an absence of data to define what is ‘safe’, the decision is best made by weighing the level of contact against the type of implants used, extent of the fusion, and time since surgery” ([2], p. 30).

Published studies for the return to physical activity and sports have a focus on surgeon recommendations rather than measuring when patients are actually returning to activities [6]. For patients who have had surgery to correct scoliosis and are returning to physical activities: “the gap between what physicians feel is appropriate for patients and what patients actually do is a fertile area for further study” ([4], p. 34). In a recent study which addressed this gap, researchers found that patients often returned to activities much earlier than recommended [6]. In another study that looked at late return to activities, the researchers dichotomized the activity variable as “part time” activity (non-contact but more than walking) and “full time” activity (contact, unrestricted) [8], and found that patients did not return to physical activity later than expected [8].

The purpose of this project was to synthesize the literature on returning to physical activities following pediatric scoliosis surgery to make it more accessible to clinicians, and to develop and test a questionnaire to explore what activities children are participating in following back surgery, and how long they are waiting before returning to specific activities.

## Main text

### Methods

This study initially involved synthesizing the literature on return to physical activities following pediatric scoliosis surgery. We then developed a mixed methods study with a quantitative questionnaire and qualitative interviews, and asked post-operative patients whether/when they were engaging in activities and compared this with the guidelines. We also asked whether they understood surgeon recommendations, and asked them to reflect on barriers to participation in sport and activities, and challenges to adherence with recommendations.

Inclusion criteria included male or female patients aged 10–18 years with a diagnosis of idiopathic scoliosis who had received posterior instrumentation and spinal fusion surgery within two years prior to the study. All clinic patients who met inclusion criteria at the time of the study (approximately thirty patients) were invited through email or in person to participate, through the scoliosis clinic research coordinator. Data collection involved a questionnaire administered through Survey Monkey. No incentives were offered.

#### Materials

A questionnaire was developed for this project to assess how soon participants engaged in activities following surgery. The questionnaire was developed based on the checklist given to patients following surgery at the scoliosis clinic, the literature review, and a list of “typical activities of childhood in four categories (personal care, school/productivity, hobbies/social activities, and sports)” based on The Paediatric Activity Card Sort [9]. The questionnaire also included questions on lifting objects of various weights and divided sports into light non-contact sports, non-contact sports with a risk of falling, contact sports, collision sports, and rotational sports. The definition of contact and collision sports was obtained from a study in which researchers assessed return to sports following shoulder surgery [10]. The questionnaire is included in Additional file 1.

Participants were asked which activities they did and were given options for when they engaged in those activities post-surgically. This was presented as a grid with radio buttons, with an opportunity for additional comments throughout. Participants were also asked to report if they engaged in any additional activities or sports not on the questionnaire, and whether they did the sports competitively*.*

Participants were invited to participate in an interview; these were conducted by telephone. Interview questions included asking participants:

- Whether they understood the guidelines they had been given
- How they felt about their activities being limited
- Whether they returned to activities they had done before their surgery
- Whether there were activities that were no longer allowed that they missed
- Whether there were activities missing in the guidelines that they were unsure about (and whether they engaged in these activities)

Interviews were audio recorded, transcribed, and analyzed deductively by question [11].

Eight patients (4 m, 4 f) were recruited and completed the questionnaire. Participants were ages 14–17 (M = 15.4) at the time of study participation. Age of onset for these participants ranged from 7 to 14 years (M = 11.5). Three of these participants also completed a 10-min follow-up interview.

### Results

There is no consensus in the literature regarding return to activity following scoliosis surgery. We compiled the existing literature on guidelines for patients following scoliosis surgery, developed a comprehensive questionnaire for activities and participation including sports to determine what and when patients engaged in activities post-surgery and pilot tested the questionnaire and interview guide [11].

#### Integration of literature on activity and participation guidelines

Studies indicate variable guidelines for return to physical activities. These studies are presented in Table 1, organized by earliest recommended activities to latest.

**Table 1:**
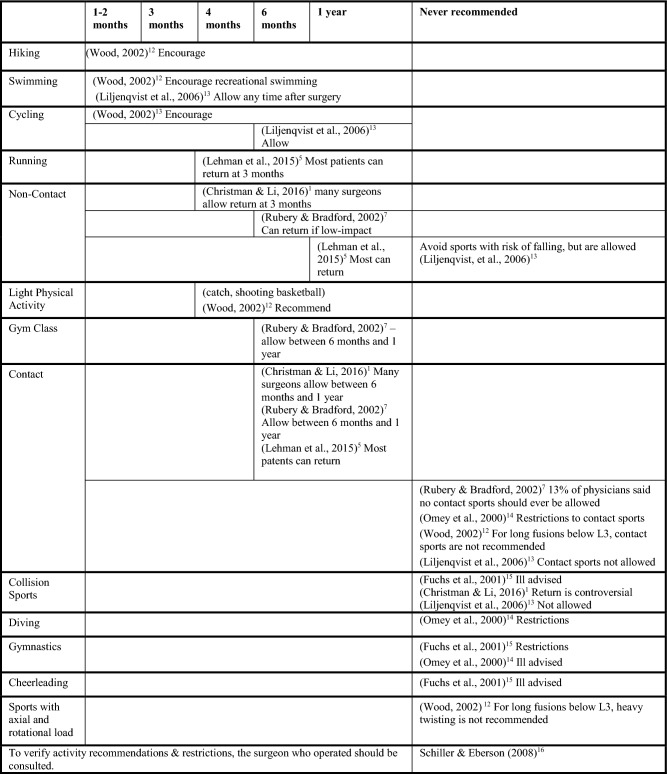
Literature-based recommendations for return to activities, organized by activity

#### Questionnaire results

Sixty-three percent of respondents (5/8) engaged in at least one physical activity before the post-operative guidelines recommended; 75% of respondents (6/8) engaged in at least one physical activity later than the post-operative guidelines recommended. Table 2 breaks this out by activity type and specific activity.

**Table 2:** Percentage of participants engaging in activities early, later than recommended, or when recommended

Activity	Percentage of Participants reporting engagement in activity	Percentage started activity before recommended in literature	Percentage started at time recommended	Percentage started after recommended in literature
Self-Care/Daily Living (shower, bath, independent self-care, chores)	100% (8/8)		100% (8/8)	
School	100% (8/8)		100% (8/8)	
Leisure Activities (reading/videogames)	75% (6/8)		83% (5/6)	16% (1/6)
Light non-contact	100% (8/8)			
Swimming			37% (3/8)	37% (3/8)
Catching/Shooting/Light Jogging		12% (1/8)	50% (4/8)	25% (2/8)
Stationary Bike				25% (2/8)
Yoga/Stretching		12% (1/8)		37% (3/8)
Running		12% (1/8)		50% (4/8)
Cycling				12% (1/8)
Non-contact with risk of falling	75% (6/8)			
Skiing/Snowboarding		16% (1/6)	33% (2/6)	
Skating		16% (1/6)	50% (3/6)	
Longboard/skateboard		16% (1/6)		
Mountain Biking			16% (1/6)	
Contact Sports	50% (4/8)			
Basketball/Volleyball			75% (3/4)	
Soccer		25% (1/4)		
Collision Sports	25% (2/8)			
Hockey/lacrosse		50% (1/2)	50% (1/2)	
Martial Arts		50.00% (1/2)		
Rotational Activities	62% (5/8)			
Golf		20% (1/5)		
Trampoline		40% (3/5)		
Cheerleading/Dance		20% (1/5)		
Softball		20% (1/5)		
Weights (0–20 +)	100% (8/8)			
0–5 Lbs			75% (6/8)	25% (2/8)
5–10 Lbs		25% (2/8)	50% (4/8)	12% (1/8)
20 + Lbs		25% (2/8)		37% (3/8)

Cells without data are omitted in this table

The eight participants reported engaging in 87 activities. We have included adherence to self-care, school and leisure guidelines, and we have compared these with our clinic guidelines; all other engagement responses were based on comparisons with the literature. Twenty-eight percent of the activities indicated early engagement, 24% were after the recommended time, and 48% were in accordance with the literature guidelines. When isolating physical activity (excluding self-care and daily living), our participants reported engaging in 65 activities. Thirty-seven percent of the responses indicated engagement before recommendations, 31% indicated engagement after the recommended time, and 32% were in accordance with the guidelines. The raw data are reported in Additional file 2.

#### Interview results

All three interviewees reported having a clear understanding of the guidelines and had a basic understanding of the possible complications if they did not adhere to the guidelines. All participants reported that their guidelines were easily accessible and that there was a copy posted at home and they carried the guidelines around with them on their phones. The process of deciding whether to do an activity included steps: *“I talk to my mom and my dad but then also, if I’m doing something and its hurting or whatever I just stop. Or if I’m not really too sure about something, I’ll do a little bit… or lift a portion of it if that’s possible, and then if I’m okay I just kind of keep going and then if it hurts, I stop immediately.”* Pain led to stopping an activity, and fear was a factor that created avoidance behaviour and kept participants from trying allowed activities: *“I’m really scared about getting hurt.”*

When asked about how they felt not being able to participate in sports and activity for a period of time after surgery, participants reported feeling frustrated, and reported that they missed playing on their sports teams: *“It sucked for a while; I was a pretty active person so the first 6–7 months kind of sucked”.* Participants reported particularly missing the social interaction that comes with sports: *“I used to work out at lunch with my friends and I can’t do that anymore…it’s just sucky”.*

### Discussion

Physical activity is an important factor in development, physically, mentally, and socially. For children recovering from scoliosis surgery, it can be difficult to participate in sports due to the presence of pain and decrease in flexibility [2], and it can be difficult for caregivers to direct patients to activities that are safe and would not put them at risk of complications. A comprehensive set of guidelines would allow surgeons to determine adherence, as well as discern possible factors influencing the return to sport.

To the current researchers’ knowledge, this is the first study to analyze the participation of physical activity following spinal surgery and compare those findings with the guidelines found in literature as well as those laid out by surgeons to determine adherence. A similar study tracked the return to physical activity and sports but did not compare their findings with any specific guidelines from the literature [6]. Another study compared participants’ activity with guidelines, but only measured return to activity in terms of part time or full time rather than measuring what kinds of activities participants were doing [8].

Although the guidelines for post-operative participation in sports provided to our participants by their surgeons were reported to be clear and easy to understand by all participants, this understanding was not reflected in participant adherence. Non-adherence to recommended activities means two different things. Firstly, children may be engaging too early in activities. This is the more critical form of non-adherence because of the risk of injury. In our sample, there were some risky activities reported including trampolining at 2 months, longboarding/skateboarding and skating at 6 months, and skiing, playing soccer, and playing hockey/lacrosse at 10 months. A second type of non-adherence is when children fail to engage in activities that they could be doing. This is less likely to result in injury, but leaves the child missing potentially beneficial activities.

The return to sports before the recommended time in our participants appears to be related to emotional and social factors: frustration and social withdrawal. The return to activities later than participants could return may be related to medical and emotional factors: pain and fear.

Due to the social implications resulting from withdrawing from sport and the findings that indicated that social withdrawal may influence an early return to sport, further research is needed on the effects that social needs have on the return to sport following surgery, particularly for children and adolescents. Recommendations by surgeons could be adapted to foresee an active patient’s difficulties in not being able to be involved in sports and suggest ways of being included in sports teams without participating in unsafe activities.

Further research is needed on the impact that rotational sports may have following spinal surgery as nearly all participants who engaged in those activities participated earlier than recommended, and some literature recommends not allowing rotational sports at all. Additionally, there are some activities for which there are no published recommendations: activities such as fishing, martial arts, yoga, softball, and mountain biking (which might be quite different than urban cycling).

## Limitations

The most significant limitation of the current research is its small sample size. It is an additional but related limitation that recruitment was limited to one facility. Primary contact with potential participants was through email correspondence which can limit participation. Use of Survey Monkey resulted in a tedious consent process as the parents had to first consent and then the child needed to assent: an onsite or in-person consent process may have resulted in more participants. Additionally, a question on patient ethnicity and/or race could be added to the questionnaire, as this has been found to be related to recovery [17, 18] and may be related to whether a patient feels well enough to engage in activities post-surgery. In addition, further lifestyle questions that can affect rehabilitation could be asked.

## Supplementary Information

**Additional file 1. **Survey Monkey Questionnaire.

**Additional file 2. **Raw Data.

## Data Availability

All data generated or analysed during this study are included in this published article and its supplementary information files. The raw data are included in Additional file 2.

## Abbreviation

AIS: Adolescent idiopathic scoliosis
