# Supplementary material for: Timelines for returning to physical activity following pediatric spinal surgery: recommendations from the literature and preliminary data
Source: BMC Res Notes. 2021 Apr 29;14:159. doi: 10.1186/s13104-021-05571-2 (PMC8082610; doi:10.1186/s13104-021-05571-2)
Supplement: Supplementary file 1 — Additional file 1. Survey Monkey Questionnaire. [file 13104_2021_5571_MOESM1_ESM.pdf]

Additional File 1: Survey Monkey Questionnaire

* When did you have your surgery?

Date

Date

MM/DD/YYYY

* How old are you?

What is your gender

- Male
- Female
- Other/Prefer not to answer

Participation in activities at home and in the community following surgery for back conditions

Since your surgery...

Do you shower?

- ◯ Yes
- ◯ No
- ◯ I don't know

How long after your surgery did you start showering?

- ◯ Immediately post surgery
- ◯ 1 month
- ◯ 6 weeks
- ◯ 2 months
- ◯ 3 months
- ◯ 4 months
- ◯ 5 months
- ◯ 6 months
- ◯ 7 months
- ◯ 8 months
- ◯ 9 months
- ◯ 10 months
- ◯ 11 months
- ◯ 1 year
- ◯ 2 years
- ◯ 3 years

Do you plan to shower in the future?

- ◯ Yes
- ◯ No
- ◯ I don't know

Why do you not do this activity now?

Do you take a bath?

- ◯ Yes
- ◯ No
- ◯ I don't know

How long after your surgery did you start taking a bath?

- ◯ Immediately post surgery
- ◯ 1 month
- ◯ 6 weeks
- ◯ 2 months
- ◯ 3 months
- ◯ 4 months
- ◯ 5 months
- ◯ 6 months
- ◯ 7 months
- ◯ 8 months
- ◯ 5 months
- ◯ 6 months
- ◯ 7 months
- ◯ 8 months
- ◯ 9 months
- ◯ 10 months
- ◯ 11 months
- ◯ 1 year
- ◯ 2 years
- ◯ 3 years

Do you plan to take a bath in the future?

- ◯ Yes
- ◯ No
- ◯ I don't know

Why do you not do this activity now?

Are you independent for all of your self-care e.g. dressing?

- Yes
- No
- I don't know

How long after your surgery did you become independent for all of your self-care?

- Immediate post surgery
- 1 month
- 6 weeks
- 2 months
- 3 months
- 4 months
- 5 months
- 6 months
- 7 months
- 8 months
- 9 months
- 10 months
- 11 months
- 1 year
- 2 years
- 3 years

Do you plan to become independent for all of your self-care?

- Yes
- No
- I don't know

Why do you not do this activity now?

Do you go to school?

- ○ Yes
- ○ No
- ○ I don't know

How long after your surgery did you go to school?

- ○ Immediately post surgery
- ○ 1 month
- ○ 6 weeks
- ○ 2 months
- ○ 3 months
- ○ 4 months
- ○ 5 months
- ○ 6 months
- ○ 7 months
- ○ 8 months
- ○ 9 months
- ○ 10 months
- ○ 11 months
- ○ 1 year
- ○ 2 years
- ○ 3 years

Do you plan to go to school in the future?

- ○ Yes
- ○ No
- ○ I don't know

Why do you not do this activity now?

Do you do chores?

- Yes
- No
- I don't know

How long after your surgery did start doing chores?

- Immediately post surgery
- 1 month
- 6 weeks
- 2 months
- 3 months
- 4 months
- 5 months
- 6 months
- 7 months
- 8 months
- 9 months
- 10 months
- 11 months
- 1 year
- 2 years
- 3 years

Do you plan to do chores in the future?

- Yes
- No
- I don't know

Why do you not do this activity now?

Do you go swimming?

- ◯ Yes
- ◯ No
- ◯ I don't know

How long after your surgery did start swimming?

- ◯ Immediately post surgery
- ◯ 1 month
- ◯ 6 weeks
- ◯ 2 months
- ◯ 3 months
- ◯ 4 months
- ◯ 5 months
- ◯ 6 months
- ◯ 7 months
- ◯ 8 months
- ◯ 9 months
- ◯ 10 months
- ◯ 11 months
- ◯ 1 year
- ◯ 2 years
- ◯ 3 years

Do you plan to go swimming in the future?

- ◯ Yes
- ◯ No
- ◯ I don't know

Why do you not do this activity now?

Do you do/prefer any of these activities: reading, video games, or board games?

- Yes
- No
- I don't know

Comments?

How long after your surgery did you start doing any of these activities: reading, video games, or board games?

- Immediately post surgery
- 1 month
- 6 weeks
- 2 months
- 3 months
- 4 months
- 5 months
- 6 months
- 7 months
- 8 months
- 9 months
- 10 months
- 11 months
- 1 year
- 2 years
- 3 years

Do you plan to do any of these activities (reading, video games, or board games) in the future?

- Yes
- No
- I don't know

Why do you not do this activity now?

Do you participate in boxing?

- Yes
- No
- I don't know

How long after your surgery did you start participating in boxing?

- Immediately post surgery
- 1 month
- 6 weeks
- 2 months
- 3 months
- 4 months
- 5 months
- 6 months
- 7 months
- 8 months
- 9 months
- 10 months
- 11 months
- 1 year
- 2 years
- 3 years

Do you plan to participate in boxing in the future?

- Yes
- No
- I don't know

Why do you not do this activity now?

Do you do any type of martial arts?

- Yes
- No
- I don't know

Comments?

How long after your surgery did you start doing any type of martial arts?

- Immediately post surgery
- 1 month
- 6 weeks
- 2 months
- 3 months
- 4 months
- 5 months
- 6 months
- 7 months
- 8 months
- 9 months
- 10 months
- 11 months
- 1 year
- 2 years

Do you plan to do any type of martial arts in the future?

- Yes
- No
- I don't know

Why do you not do this activity now?

Do you do any of the following: catch, shoot basketballs, or light jogging?

- Yes
- No

How long after your surgery did you start doing any of the following: catch, shoot basketballs, or light jogging?

- Immediately post surgery
- 1 month
- 6 weeks
- 2 months
- 3 months
- 4 months
- 5 months
- 6 months
- 7 months
- 8 months
- 9 months
- 10 months
- 11 months
- 1 year
- 2 years

Do you plan to do any of of the following activities in the future: catch, shoot basketballs, or light jogging?

- Yes
- No
- I don't know

Why do you not do this activity now?

Do you do any of the following: golf or tennis?

- Yes
- No
- I don't know

Comments?

How long after your surgery did you start doing any of the following: golf or tennis?

- Immediately post surgery
- 1 month
- 6 weeks
- 2 months
- 3 months
- 4 months
- 5 months
- 6 months
- 7 months
- 8 months
- 9 months
- 10 months
- 11 months
- 1 year
- 2 years
- 3 years

Do you plan to do any of of the following activities in the future: golf or tennis?

- Yes
- No

Why do you not do this activity now?

Do you use a stationary bike?

- Yes
- No
- I don't know

How long after your surgery did you start using a stationary bike?

- Immediately post surgery
- 1 month
- 6 weeks
- 2 months
- 3 months
- 4 months
- 5 months
- 6 months
- 7 months
- 8 months
- 9 months
- 10 months
- 11 months
- 1 year
- 2 years
- 3 years

Do you plan to use a stationary bike in the future?

- Yes
- No
- I don't know

Why do you not do this activity now?

Do you do any of the following: yoga, stretching, or Pilates?

- Yes
- No
- I don't know

Comments?

How long after your surgery did you start doing any of these activities: yoga, stretching, or Pilates?

- Immediately post surgery
- 1 month
- 6 weeks
- 2 months
- 3 months
- 4 months
- 5 months
- 6 months
- 7 months
- 8 months
- 9 months
- 10 months
- 11 months
- 1 year
- 2 years
- 3 years

Do you plan to do any of the following activities in the future: yoga, stretching, or Pilates?

- Yes
- No
- I don't know

Why do you not do this activity now?

Do you do any of the following: cheerleading or dance?

- Yes
- No
- I don't know

Comments?

How long after your surgery did you start doing any of these activities: cheerleading or dance?

- Immediately post surgery
- 1 month
- 6 weeks
- 2 months
- 3 months
- 4 months
- 5 months
- 6 months
- 7 months
- 8 months
- 9 months
- 10 months
- 11 months
- 1 year
- 2 years
- 3 years

Do you plan to do any of the following activities in the future: cheerleading or dance?

- Yes
- No
- I don't know

Why do you not do this activity now?

Do you do gymnastics?

- Yes
- No
- I don't know

Comments?

How long after your surgery did you start doing gymnastics?

- Immediately post surgery
- 1 month
- 6 weeks
- 2 months
- 3 months
- 4 months
- 5 months
- 6 months
- 7 months
- 8 months
- 9 months
- 10 months
- 11 months
- 1 year
- 2 years
- 3 years

Do you plan to do gymnastics in the future?

- Yes
- No
- I don't know

Why do you not do this activity now?

Do you run?

- Yes
- No
- I don't know

How long after your surgery did you start running?

- Immediately post surgery
- 1 month
- 6 weeks
- 2 months
- 3 months
- 4 months
- 5 months
- 6 months
- 7 months
- 8 months
- 9 months
- 10 months
- 11 months
- 1 year
- 2 years
- 3 years

Do you plan to run in the future?

- Yes
- No
- I don't know

Why do you not do this activity now?

Do you go skating?

- ◯ Yes
- ◯ No
- ◯ I don't know

How long after your surgery did you start skating?

- ◯ Immediately post surgery
- ◯ 1 month
- ◯ 6 weeks
- ◯ 2 months
- ◯ 3 months
- ◯ 4 months
- ◯ 5 months
- ◯ 6 months
- ◯ 7 months
- ◯ 8 months
- ◯ 9 months
- ◯ 10 months
- ◯ 11 months
- ◯ 1 year
- ◯ 2 years
- ◯ 3 years

Do you plan to go skating in the future?

- ◯ Yes
- ◯ No
- ◯ I don't know

Why do you not do this activity now?

Do you go Skiing/Snowboarding?

- ◯ Yes
- ◯ No
- ◯ I don't know

How long after your surgery did you start Skiing/Snowboarding?

- ◯ Immediately post surgery
- ◯ 1 month
- ◯ 6 weeks
- ◯ 2 months
- ◯ 3 months
- ◯ 4 months
- ◯ 5 months
- ◯ 6 months
- ◯ 7 months
- ◯ 8 months
- ◯ 9 months
- ◯ 10 months
- ◯ 11 months
- ◯ 1 year
- ◯ 2 years
- ◯ 3 years

Do you plan to go Skiing/Snowboarding in the future?

- ◯ Yes
- ◯ No
- ◯ I don't know

Why do you not do this activity now?

[ ]

Do you go curling?

- ◯ Yes
- ◯ No
- ◯ I don't know

How long after your surgery did you start curling?

- ◯ Immediately post surgery
- ◯ 1 month
- ◯ 6 weeks
- ◯ 2 months
- ◯ 3 months
- ◯ 4 months
- ◯ 5 months
- ◯ 6 months
- ◯ 7 months
- ◯ 8 months
- ◯ 9 months
- ◯ 10 months
- ◯ 11 months
- ◯ 1 year
- ◯ 2 years
- ◯ 3 years

Do you plan to go curling in the future?

- ◯ Yes
- ◯ No
- ◯ I don't know

Why do you not do this activity now?

Do you go cycling?

- ◯ Yes
- ◯ No
- ◯ I don't know

How long after your surgery did you start cycling?

- ◯ Immediately post surgery
- ◯ 1 month
- ◯ 6 weeks
- ◯ 2 months
- ◯ 3 months
- ◯ 4 months
- ◯ 5 months
- ◯ 6 months
- ◯ 7 months
- ◯ 8 months
- ◯ 9 months
- ◯ 10 months
- ◯ 11 months
- ◯ 1 year
- ◯ 2 years
- ◯ 3 years

Do you plan to go cycling in the future?

- ◯ Yes
- ◯ No
- ◯ I don't know

Why do you not do this activity now?

Do you go mountain biking?

- Yes
- No
- I don't know

How long after your surgery did you start mountain biking?

- Immediately post surgery
- 1 month
- 6 weeks
- 2 months
- 3 months
- 4 months
- 5 months
- 6 months
- 7 months
- 8 months
- 9 months
- 10 months
- 11 months
- 1 year
- 2 years
- 3 years

Do you plan to go mountain biking in the future?

- Yes
- No
- I don't know

Why do you not do this activity now?

Do you use a Skateboard/Long Board/Scooter?

- Yes
- No
- I don't know

How long after your surgery did you start using a Skateboard/Long Board/Scooter?

- Immediately post surgery
- 1 month
- 6 weeks
- 2 months
- 3 months
- 4 months
- 5 months
- 6 months
- 7 months
- 8 months
- 9 months
- 10 months
- 11 months
- 1 year
- 2 years
- 3 years

Do you plan to use a Skateboard/Long Board/Scooter in the future?

- Yes
- No
- I don't know

Why do you not do this activity now?

Do you play soccer?

- Yes
- No
- I don't know

How long after your surgery did you start playing soccer?

- Immediately post surgery
- 1 month
- 6 weeks
- 2 months
- 3 months
- 4 months
- 5 months
- 6 months
- 7 months
- 8 months
- 9 months
- 10 months
- 11 months
- 1 year
- 2 years
- 3 years

Do you plan to play soccer in the future?

- Yes
- No
- I don't know

Why do you not do this activity now?

Do you play basketball or volleyball?

- Yes
- No
- I don't know

How long after your surgery did you start playing basketball or volleyball?

- Immediately post surgery
- 1 month
- 6 weeks
- 2 months
- 3 months
- 4 months
- 5 months
- 6 months
- 7 months
- 8 months
- 9 months
- 10 months
- 11 months
- 1 year
- 2 years
- 3 years

Do you plan to play basketball or volleyball in the future?

- Yes
- No
- I don't know

Why do you not do this activity now?

Do you go on the trampoline?

- ◯ Yes
- ◯ No
- ◯ I don't know

How long after your surgery did you start going on the trampoline?

- ◯ Immediately post surgery
- ◯ 1 month
- ◯ 6 weeks
- ◯ 2 months
- ◯ 3 months
- ◯ 4 months
- ◯ 5 months
- ◯ 6 months
- ◯ 7 months
- ◯ 8 months
- ◯ 9 months
- ◯ 10 months
- ◯ 11 months
- ◯ 1 year
- ◯ 2 years
- ◯ 3 years

Do you plan to go on the trampoline in the future?

- ◯ Yes
- ◯ No
- ◯ I don't know

Why do you not do this activity now?

Do you go horseback riding?

- ◯ Yes
- ◯ No
- ◯ I don't know

How long after your surgery did you start horseback riding?

- ◯ Immediately post surgery
- ◯ 1 month
- ◯ 6 weeks
- ◯ 2 months
- ◯ 3 months
- ◯ 4 months
- ◯ 5 months
- ◯ 6 months
- ◯ 7 months
- ◯ 8 months
- ◯ 9 months
- ◯ 10 months
- ◯ 11 months
- ◯ 1 year
- ◯ 2 years
- ◯ 3 years

Do you plan to go horseback riding in the future?

- ◯ Yes
- ◯ No
- ◯ I don't know

Why do you not do this activity now?

Do you wrestle?

- Yes
- No
- I don't know

How long after your surgery did you start wrestling?

- Immediately post surgery
- 1 month
- 6 weeks
- 2 months
- 3 months
- 4 months
- 5 months
- 6 months
- 7 months
- 8 months
- 9 months
- 10 months
- 11 months
- 1 year
- 2 years
- 3 years

Do you plan to wrestle in the future?

- Yes
- No
- I don't know

Why do you not do this activity now?

Do you play football or rugby?

- Yes
- No
- I don't know

Comments?

How long after your surgery did you start playing football or rugby?

- Immediately post surgery
- 1 month
- 6 weeks
- 2 months
- 3 months
- 4 months
- 5 months
- 6 months
- 7 months
- 8 months
- 9 months
- 10 months
- 11 months
- 1 year
- 2 years
- 3 years

Do you plan to play football or rugby in the future?

- Yes
- No
- I don't know

Why do you not do this activity now?

Do you play hockey or lacrosse?

- Yes
- No
- I don't know

Comments?

How long after your surgery did you start playing hockey or lacrosse?

- Immediately post surgery
- 1 month
- 6 weeks
- 2 months
- 3 months
- 4 months
- 5 months
- 6 months
- 7 months
- 8 months
- 9 months
- 10 months
- 11 months
- 1 year
- 2 years
- 3 years

Do you plan to play hockey or lacrosse in the future?

- Yes
- No
- I don't know

Why do you not do this activity now?

Do you participate in rodeo sports?

- ◯ Yes
- ◯ No
- ◯ I don't know

Comments?

How long after your surgery did you start participating in rodeo sports?

- ◯ Immediately post surgery
- ◯ 1 month
- ◯ 6 weeks
- ◯ 2 months
- ◯ 3 months
- ◯ 4 months
- ◯ 5 months
- ◯ 6 months
- ◯ 7 months
- ◯ 8 months
- ◯ 9 months
- ◯ 10 months
- ◯ 11 months
- ◯ 1 year
- ◯ 2 years
- ◯ 3 years

Do you plan to participate in rodeo sports in the future?

- ◯ Yes
- ◯ No
- ◯ I don't know

Why do you not do this activity now?

Do you lift things that weigh 0-5 pounds?

- ◯ Yes
- ◯ No
- ◯ I don't know

How long after your surgery did you start lift anything that weighs 0-5 pounds?

- ◯ Immediately post surgery
- ◯ 1 month
- ◯ 6 weeks
- ◯ 2 months
- ◯ 3 months
- ◯ 4 months
- ◯ 5 months
- ◯ 6 months
- ◯ 7 months
- ◯ 8 months
- ◯ 9 months
- ◯ 10 months
- ◯ 11 months
- ◯ 1 year
- ◯ 2 years
- ◯ 3 years

Do you plan to lift something that weighs 0-5 pounds in the future?

- ◯ Yes
- ◯ No
- ◯ I don't know

Why do you not do this activity now?

Do you lift things that weigh 5-10 pounds?

- Yes
- No
- I don't know

How long after your surgery did you start lifting things that weigh 5-10 pounds?

- Immediately post surgery
- 1 month
- 6 weeks
- 2 months
- 3 months
- 4 months
- 5 months
- 6 months
- 7 months
- 8 months
- 9 months
- 10 months
- 11 months
- 1 year
- 2 years
- 3 years

Do you plan to lift things that weigh 5-10 pounds in the future?

- Yes
- No
- I don't know

Why do you not do this activity now?

Do you lift things that weigh 20 pounds or more?

- ○ Yes
- ○ No
- ○ I don't know

How long after your surgery did you start lifting things that weigh 20 pounds or more?

- ○ Immediately post surgery
- ○ 1 month
- ○ 6 weeks
- ○ 2 months
- ○ 3 months
- ○ 4 months
- ○ 5 months
- ○ 6 months
- ○ 7 months
- ○ 8 months
- ○ 9 months
- ○ 10 months
- ○ 11 months
- ○ 1 year
- ○ 2 years
- ○ 3 years

Do you plan to lift things that weigh 20 pounds or more in the future?

- ○ Yes
- ○ No
- ○ I don't know

Why do you not do this activity now?

[ ]

Do you do any other activity, game, or sport that we haven't asked about?

- ◯ Yes
- ◯ No
- ◯ I don't know

Please type in all other activities you have been doing, and (after each activity) how long after surgery you started doing those activities? (Example: Flag Football (4 months after Surgery)

Are there activities that you don't do but would like to do?

- ◯ Yes
- ◯ No
- ◯ I don't know

If yes, please specify:

Why do you not do them?

Do you play sports competitively?

- Yes
- No
- I don't know

Which sports do you play competitively?

How long after your surgery did you start doing these activities?

- Immediately post surgery
- 1 month
- 6 weeks
- 2 months
- 3 months
- 4 months
- 5 months
- 6 months
- 7 months
- 8 months
- 9 months
- 10 months
- 11 months
- 1 year
- 2 years
- 3 years

Do you plan to play sports competitively in the future?

- Yes
- No
- I don't know

Why do you not do this activity now?

Was the information on activity suggestions given to you from the clinic following surgery easy to understand?

- Yes
- No

Why or why not?

Would you like to participate in a more in-depth interview (15 minutes in the fall) to helps us gain greater insight into your responses?

- Yes
- No

Please provide your name so we can contact you for the interview:

Please provide your phone number or email address:

Those are all of the questions we have for you right now. Thank you so much for answering our questions.

Title of Research Study:
Participation in Activities at Home and in the Community Following Surgery for Back Conditions
