# Supplementary material for: Timelines for returning to physical activity following pediatric spinal surgery: recommendations from the literature and preliminary data
Source: BMC Res Notes. 2021 Apr 29;14:159. doi: 10.1186/s13104-021-05571-2 (PMC8082610; doi:10.1186/s13104-021-05571-2)
Supplement: Supplementary file 2 — Additional file 2. Raw Data. [file 13104_2021_5571_MOESM2_ESM.pdf]

Additional File 2: Raw Data

Activity	Immediately	1 month	2 months	3 months	4 months	6 months	7 months	8 months	10 months	12 months
**Shower**	**6**	**2**								
**Independent Self-Care**	**2**	**4**			**1**					
**Bath**		**1**	**3**							
**Chores**		**2**	**4**	**1**						
**School**		**4**	**4**							
**Reading/Videogames**	**5**	**1**		**1**						
**Swimming**			**1**	**1**	**1**	**3**				
**Stationary Bike**					**1**	**1**				
**Catching/Shooting/light Jogging**				**2**	**2**					**2**
**Cycling *Assume Light Non-Contact***										**1**
**Yoga/Stretching**	**1**				**1**	**1**				**1**
**Running**			**1**		**1**	**2**				**1**
**Skiing/Snowboarding**									**1**	**1**
**Skating**						**1**				**3**
**Long Boarding/Skateboarding**						**1**				**1**
**Mountain Biking**										**1**
**Basketball/Volleyball**								**1**		**2**
**Soccer (Contact)**									**1**<br>*** surgeon approval**	
**Hockey/Lacrosse (Contact)**									**1**	**1**
**Gymnastics**										
**Trampoline**			**1**							**2**
**Martial Arts**				**1**						
**Golf/Tennis**						**1**	**1**			
**Soft ball**						**1**				
**Fishing**			**1**							
**Lift 0-5 lbs**		**6**	**1**	**1**						
**Lift 5-10 lbs**			**3**	**1**	**1**	**2**				
**Lift 20+ lbs**				**2**				**1**		**2**
